# Short-term power load forecasting method based on Bagging-stochastic configuration networks

**DOI:** 10.1371/journal.pone.0300229

**Published:** 2024-03-19

**Authors:** Xinfu Pang, Wei Sun, Haibo Li, Wei Liu, Changfeng Luan

**Affiliations:** 1 Key Laboratory of Energy Saving and Controlling in Power System of Liaoning Province, Shenyang Institute of Engineering, Shenyang, Liaoning, China; 2 Yingkou Power Supply Company, State Grid Liaoning Electric Power Co., Ltd., Yingkou, Liaoning, China; Newcastle University, UNITED KINGDOM

## Abstract

Accurate short-term load forecasting is of great significance in improving the dispatching efficiency of power grids, ensuring the safe and reliable operation of power grids, and guiding power systems to formulate reasonable production plans and reduce waste of resources. However, the traditional short-term load forecasting method has limited nonlinear mapping ability and weak generalization ability to unknown data, and it is prone to the loss of time series information, further suggesting that its forecasting accuracy can still be improved. This study presents a short-term power load forecasting method based on Bagging-stochastic configuration networks (SCNs). First, the missing values in the original data are filled with the average values. Second, the influencing factors, such as the weather- and week-type data, are coded. Then, combined with the data of influencing factors after coding, the Bagging-SCNs integration algorithm is used to predict the short-term load. Finally, by taking the daily load data of Quanzhou City, Zhejiang Province as an example, the program of the abovementioned method is compiled in Python language and then compared with the long short-term memory neural network algorithm and the single-SCNs algorithm. Simulation results show that the proposed method for medium- and short-term load forecasting has a high forecasting accuracy and a significant effect on improving the accuracy of load forecasting.

## 1 Introduction

### 1.1 Literature review

Short-term load forecasting is the focus of people’s attention in load forecasting. In short-term load forecasting, even a 1% reduction in the average forecasting error can considerably increase the savings for utilities [1]. Highly accurate short-term load forecasting can greatly improve the economic benefits of power grids. Therefore, a short-term load forecasting model with high precision, strong stability, and strong generalization ability should be established. Furthermore, accurate short-term load forecasting is of great significance in improving the dispatching efficiency of power grids [2], ensuring the safe and reliable operation of power grids [3], guiding power systems to formulate reasonable production plans [4], and reducing the waste of resources and unnecessary economic losses [5]. At present, the methods of short-term power load forecasting are mainly divided into three categories: short-term load forecasting based on traditional statistics, short-term load forecasting based on traditional machine learning, and short-term load forecasting based on deep learning.

The idea of the short-term load forecasting method based on statistics is easy to understand; however, for nonlinear datasets, its mapping ability is weak. If new data are used as input, then they will cause large errors (i.e., poor generalization ability) in model prediction [6–8]. The short-term load forecasting model based on machine learning has a strong nonlinear mapping ability, and its generalization ability is also better compared with the traditional statistical methods, but the problem of time series information loss is serious, further suggesting that its prediction accuracy can still be improved [9–12]. In recent years, with the improvement of computing ability, deep learning has also continuously developed. Therefore, the application of deep learning in short-term power load forecasting has gradually become a research hotspot [13, 14]. The cyclic neural network offers great advantages in dealing with the problem of time series, and it can learn the change trend of the time series through the input of historical information. However, when the input time series is long, the gradient can easily disappear [15].

The methods often used in power load forecasting based on traditional statistics are the time series method, exponential smoothing method, gray model method, regression analysis method, and so on. Ref. [16] used the multilinear regression algorithm to predict high-volume power load data with a few characteristics. Ref. [17] analyzed the influencing degree of different holidays and power loads and used the Kalman filter method to predict and model different types of holidays. Ref. [18] proposed a combined load forecasting model based on the exponential smoothing method. The requirements of this method are high for power load sequences; besides, the method must meet the requirements of the small fluctuation, less random factors, and strong regularity of the load curve. For this kind of power load, the abovementioned method can quickly obtain highly accurate prediction results. However, the most prominent disadvantage of this statistical algorithm is its poor robustness. When the historical load data are nonlinear and complex, the traditional statistical algorithm can hardly achieve accurate predictions. Therefore, the ability of traditional statistical methods in dealing with nonlinear problems is poor. By contrast, machine learning algorithms are good in dealing with nonlinear problems.

Power load forecasting based on traditional machine learning mainly includes the use of artificial neural networks [19], support vector machines (SVMs), and so on. Ref. [20] proposed an improved SVM hybrid forecasting model by considering electricity price as a factor. The prediction model could extract features by using the maximum correlation and minimum redundancy method, extract the power consumption sequence during historical holidays via weighted gray correlation projection, and optimize the parameters of SVM to improve the prediction accuracy of the model. Ref. [21] proposed a short-term load forecasting method based on the wavelet transform and SVM. The time series was decomposed and input into different frequency modes, and then the wavelet kernel function was used to replace the radial basis function (RBF) kernel function. The method achieved a good forecasting effect. In Ref. [22], aimed at determining the impact of real-time electricity price on short-term load, a short-term load forecasting model was established by combining the RBF neural network (RBFNN) with the adaptive neuro-fuzzy inference system. First, the model used the nonlinear approximation ability of RBFNN to predict the load on the forecast day without considering the factors of electricity price. Then, according to the recent changes in real-time electricity price, the adaptive neuro-fuzzy inference system was used to adjust the load forecasting results of the RBFNN. This integrated system could improve the prediction accuracy and overcome the defects of RBFNNs. However, in the face of complex power big data, the useful information in the power load big data is difficult to mine, as the traditional machine learning algorithm needs to set much fewer parameters. Therefore, when the amount of data is large, the prediction results of machine learning algorithms is usually not ideal.

In recent years, prediction method combinations have been proposed and achieved good prediction results. For example, the decomposition algorithm was combined with the long-term and short-term memory neural network and gated cycle unit. The method could decompose the historical and highly nonlinear load forecasting problem into multiple nonlinear time series. Ref. [23] decomposed the wind power load sequence via variational mode decomposition to obtain three different frequency components (high, medium, and low) and then used long short-term memory (LSTM) to predict the three different frequency components respectively. Their experimental simulation showed that the accuracy of load prediction could be effectively improved. Ref. [24] combined the set empirical mode decomposition algorithm with gated recurrent units (GRU) and the multilinear regression algorithms. First, the historical load sequence was decomposed into multiple subsequences via the decomposition algorithm, and then all subsequences were divided into high- and low-frequency components via the zero-crossing rate of each subsequence. Finally, GRU and multilinear regression were used to predict the two components. The prediction accuracy of the proposed method was better than those of the common single-prediction method and the combined prediction method based on EEMD. As for the characteristic of the combined forecasting method based on sequence decomposition, it could extract the components of different frequencies in the historical load series and transform the complex historical load series forecasting problem into multiple relatively simple subsequence load forecasting problems, thus reducing the difficulty of forecasting. However, the aforementioned methods still have problems, such as difficulties in predicting the decomposed components [25], and errors are inevitable when predicting each component. Subsequently, reconstructing the predicted value of each component would lead to error accumulation, indicating the high complexity of the combined prediction model [26].

### 1.2 Motivation

Although the prediction model based on statistical theory is easy to understand, its nonlinear mapping ability is limited, and its generalization ability to unknown data is weak. The prediction model based on machine learning offers strong advantages in dealing with nonlinear problems and has strong generalization ability; however, when the amount of data is large, it is prone to loss of time series information, further suggesting that the prediction accuracy can still be improved. In contrast to the inherent shortcomings of the traditional gradient algorithm (i.e., the convergence speed is slow and easy to fall into local minimization), the stochastic configuration networks algorithm has the advantage of fast learning speed. Furthermore, the SCNs can automatically generate the corresponding network structure according to the characteristics of the input data, effectively reduce the error caused by human intervention, and improve the performance of the model. In view of the advantages of the stochastic configuration networks, a short-term load forecasting method based on the stochastic configuration networks is proposed in this study. At the same time, given the instability of the neural network itself, when the training sets are slightly different, some differences will arise in the prediction results. By integrating the neural network algorithm through Bagging, the variance of the model can be effectively reduced, and the generalization performance of the integrated model can be improved. Meanwhile, when the training data have little differences or entail noise, the output result of the integrated model will not fluctuate greatly. Thus, not only can the stability be increased, but a certain anti-noise ability can also be achieved.

On the basis of the abovementioned discussion, this study proposes a Bagging-SCNs short-term load forecasting method that takes SCNs as the basic learner, integrates multiple stochastic configuration networks by using the Bagging algorithm, and uses the integrated model for short-term load forecasting. We selected data from Quanzhou City, Zhejiang Province for the entire year of 2018 as a reference, and used load data collected every 15 minutes from January 1, 2018 to the day before the forecast date as the training set. We also used randomly selected daily load data collected every 15 minutes on June 15, June 16, December 7, and December 8 as the validation set. There were four groups in total, taking the effectiveness of the proposed method as an example.

## 2 Problem description of forecasts in short-term load forecasting

**Fig 1:**
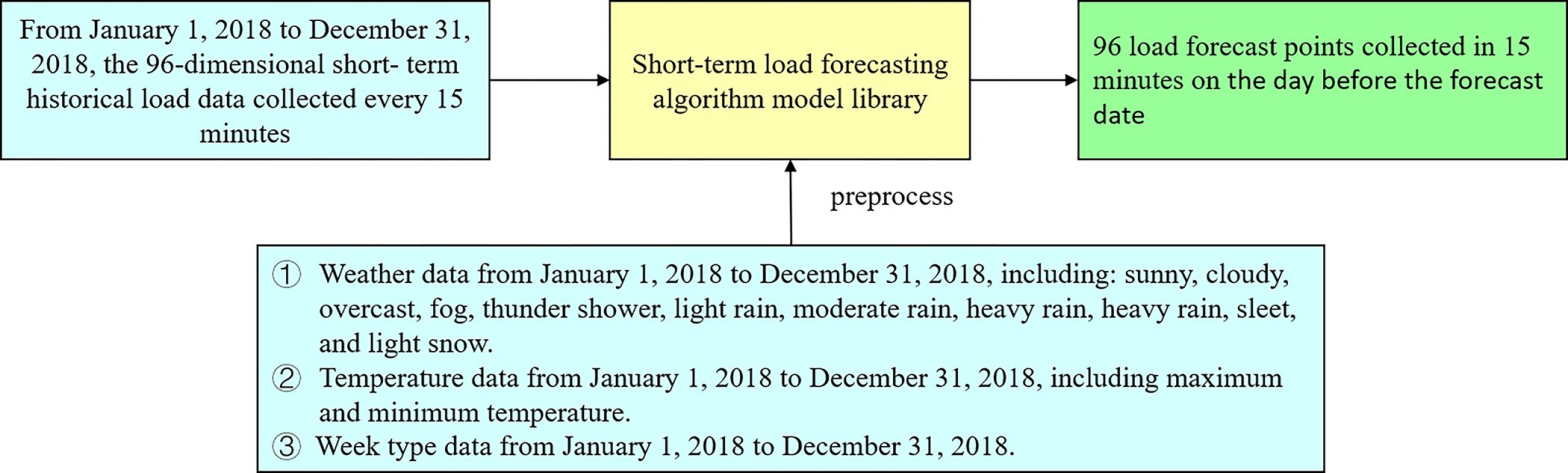
Description of the short-term load forecasting problem.

In short-term load forecasting, we should not only consider the law of load itself as changing with time but also the impact of external factors on load forecasting, such as weather, temperature (maximum, minimum, and average temperatures), and date types. According to the influencing factors of short-term load forecasting analyzed above, temperature is the most significant factor affecting short-term load forecasting, whereas weather and date types have varying degrees of influences. However, weather and date types cannot be recognized by machines during forecasting; thus, they need to be preprocessed into codes prior to machine recognition. The traditional short-term load forecasting method has a limited nonlinear mapping ability and a weak generalization ability to unknown data, and it is prone to the loss of time series information; these limitations suggest that its prediction accuracy can still be improved. Therefore, an appropriate algorithm should be selected for short-term load forecasting. The problem description of short-term load forecasting is shown in Fig 1.

## 3 Short-term load forecasting strategy structure based on Bagging-SCNs

**Fig 2:**
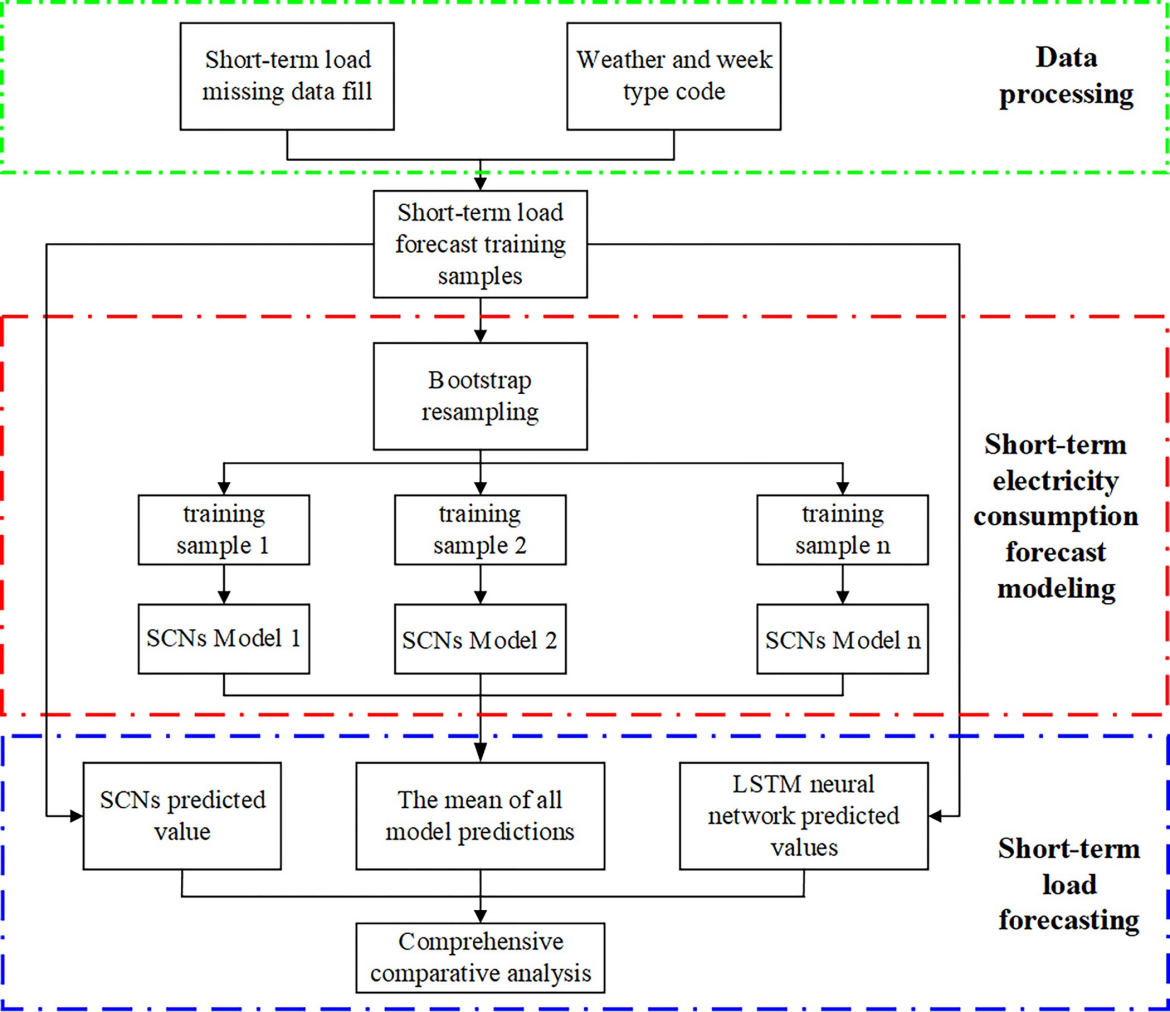
Strategy diagram of the short-term load forecasting method.

The implementation strategy of the short-term load forecasting method based on Bagging-SCNs is shown in Fig 2. The main contents of the strategy include the following: filling in the missing data of historical short-term daily load, taking the average of two values near the missing value as the filling of the missing value, encoding the weather- and week-type data, and converting the weather-type data into matrix form. The historical short-term daily load data and the influencing factor data of daily load form a training sample set, which is then resampled via the Bootstrap method to form a training subset with the same size. Then, a stochastic configuration networks model is constructed for each training subset. The average of the prediction results of all stochastic configuration networks models is taken as the final short-term load forecasting result, and then it is compared with the prediction obtained by LSTM neural network method. Finally, after the SCNs are integrated, the effectiveness of the proposed method is verified.

### 3.1 Data processing

The original short-term load data are inevitably missing; they should be filled to reduce the impact of missing data on short-term load forecasting. Examples of filling methods are zero filling, median filling, average filling, etc. In this study, the average value is used for filling. In particular, the average value of two data near the missing value is used as the filling to ensure it is as close to the real data as possible.

According to the analysis and study of the load characteristics of the existing dataset, when the model predicts the load data in the next 24 hours, other influencing factors other than the load should be determined, including the maximum temperature, minimum temperature, weather, and day of the week. The weather parameter mainly includes the following types: sunny, overcast, cloudy, fog, thunderstorm, light rain, moderate rain, heavy rain, rainstorm, sleet, and light snow. For example, when the weather changes from sunny to cloudy, sunny is represented by 1, cloudy is represented by 1, and other meteorology types are represented by 0. This dataset forms a one-dimensional matrix, e.g., [1,1,0,0,0,0,0,0,0] to represent sunny to overcast. Then, the numbers from 1 to 7 are used to represent the day of the week. For example, if the forecast day is Sunday, then it is set to 7 as the week-type data entry.

### 3.2 Modeling the short-term power consumption prediction

#### 3.2.1 Bagging integration algorithm

Ensemble learning is a general term used for the method of combining multiple learners to build a strong learner. It integrates multiple models to solve the same problem and turns it into a more powerful learner [27]. Although the specific algorithm and combination strategy of the basic learner of integrated learning differ from one another, the basic implementation steps of the integrated learning algorithm are similar. Ensemble learning first trains each basic learner through different subsets of training samples and then combines the trained basic learners by means of some combination strategy to turn a single weak learner into an integrated strong learner [28]. The integrated performance of multiple base learners is much higher than that of a single base learner [29]. At present, the mature integrated learning methods include Bagging and Boosting. In this study, the classical Bagging algorithm is used for short-term load forecasting.

**Fig 3:**
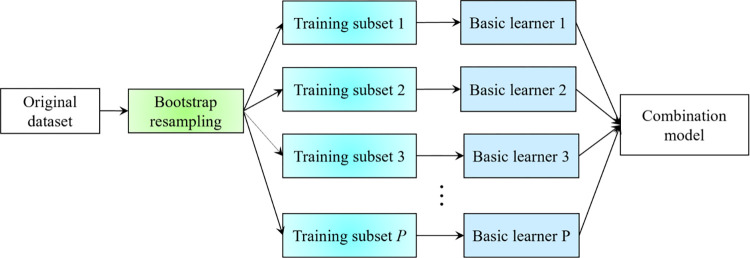
Basic flowchart of Bagging.

Breiman proposed the Bagging ensemble learning method in 1996. The basic flowchart of the Bagging method is shown in Fig 3. The flowchart depicts a parallel ensemble learning method [30]. At its core, Bagging utilizes the Bootstrap method [31], a groundbreaking resampling technique conceived by Bradley Efron in 1979, to create multiple training subsets from an original dataset. Each of these subsets retains the same sample size as the original, yet, due to the ’with-replacement’ nature of Bootstrap resampling, some observations might be frequently represented, while others might be absent entirely. Intriguingly, about 37% of the original samples tend not to be included in any given training subset and are aptly termed "out-of-bag" data. Each distinct subset serves as training grounds for a basic learner. Once trained, these learners can be synergistically combined using a specific strategy, cultivating a model that’s both robust and formidable.

For a model that uses the Bagging integration algorithm, whether its performance can be effectively improved mainly depends on the stability of the single basic learner. For unstable basic learners, i.e., for different training sets with slight differences, the output results of basic learners are highly varied, resulting in large errors in the output results of the basic learners. The common unstable basic learners are processed via decision trees and neural networks. For these unstable basic learners, the generalization ability of the model can be greatly improved and the output variance of the model can be greatly reduced after integrating the Bagging algorithm. The performance of the integrated model with the Bagging algorithm also depends on whether each base learner is independent and differs from one another. The integrated model with the Bagging algorithm can train all base learners at the same time, hence effectively saving substantial training time [32]. In addition, the basis of the Bagging algorithm is the resampling of the Bootstrap method. Before each basic learner is trained, Bootstrap is used to resample the original training set to obtain the training subset corresponding to each basic learner. The training subsets should differ from one another. Especially when neural networks and the decision tree algorithm are used as basic learners, given the instability of the algorithm itself, When the training subsets are slightly different, there will be some differences in the trained basic learners. Bagging just reduces the variance of the model by integrating each basic learner, so as to improve the generalization performance of the integrated model. When the training data are not different or there is noise, the output result of the integrated model will not fluctuate greatly and increase its stability, At the same time, it also has certain anti-noise ability.

#### 3.2.2 Stochastic configuration networks

**Fig 4:**
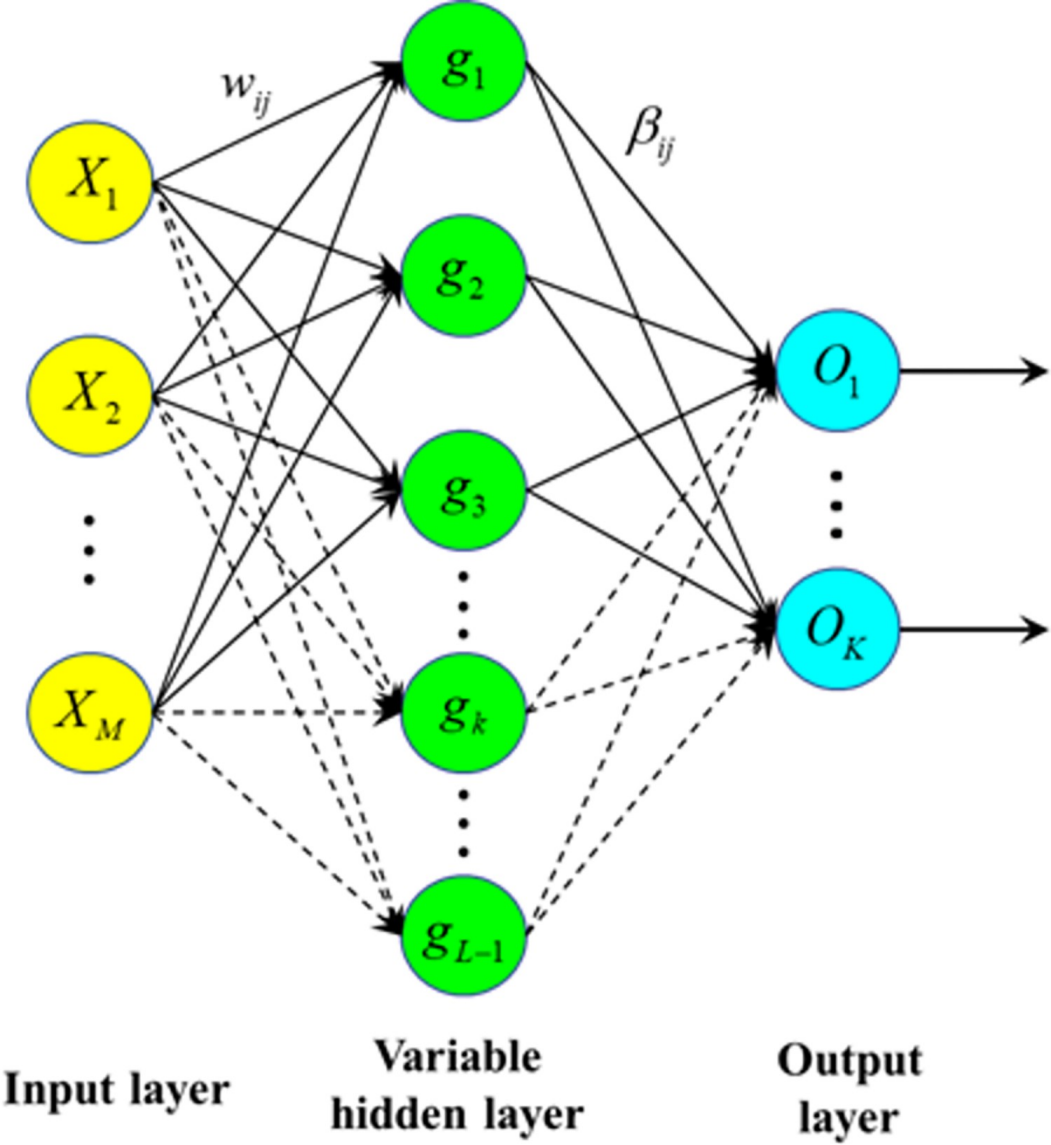
Basic network structure of SCNs.

Stochastic configuration networks (SCNs) is a supervised random weight neural network, which was proposed by Wang and Li in 2017 [33]. The network structure of SCNs is shown in Fig 4. In contrast to the traditional feedforward neural network, SCNs need less human intervention. The construction of SCNs begins with a simple foundational network, initialized with randomly selected input weights and thresholds from a predefined range, such as between -1 and 1. The output weights are initialized to values near zero, ensuring simplicity in the network’s early stages. As the network evolves, the number of neurons in the hidden layer increases, driven by the random selection of input weights and thresholds. The weights and thresholds for each network output are refined using the least squares method. Moreover, the random parameters in SCNs are not entirely arbitrary. They incorporate inequality constraints, automatically determining the suitable range for the random parameters based on the parameters already chosen. During the supervised learning process, input data is fed into the network. Using the current weights and activation functions, outputs for each layer are computed. The network then calculates the loss based on its outputs and the actual labels. This loss is differentiated to obtain gradients for each weight, which in turn are used to adjust the weights. The training ceases once the network’s accuracy reaches the predefined criteria.

The basic network structure of SCNs is shown in Fig 4. The network has the *M*−(*L*−1)−*K* structure, where *M* represents the number of neurons in the input layer, *L*−1 represents the number of neurons in the hidden layer, *K* represents the number of neurons in the output layer, and *X* is the input matrix of the stochastic configuration networks. For the short-term load forecasting adopted in this study, *X* corresponds to the daily load influencing factor data from January 1, 2018 to the day before the forecast date as the training set, including the data about temperature, coded week type, coded weather, and other factors. Thus, $X=\{X_{1},X_{2},\ldots ,X_{N}\}=[\begin{array}{cccc} x_{11} & x_{12} & \cdots & x_{1N}\\ x_{21} & x_{22} & \cdots & x_{2N}\\ \vdots & \vdots & \ddots & \vdots \\ x_{M1} & x_{M2} & \cdots & x_{MN} \end{array}]$, where *M* is the dimension of the input matrix (14 dimensions in this study), *N* is the number of samples in the input matrix (166, 167, 348, 349 samples in each of the four validation groups in this study), $X_{n}=(x_{1n},x_{2n},\ldots ,x_{Mn})$ is the *n* input sample, and $T_{n}=[t_{1n},t_{2n},\ldots ,t_{Kn}]$ is the expected output of the *n* sample, with *K* = 96 sampling points for one day of short-term daily load. $W=[\begin{array}{cccc} w_{11} & w_{12} & \cdots & w_{1L-1}\\ w_{21} & w_{22} & \cdots & w_{2L-1}\\ \vdots & \vdots & \ddots & \vdots \\ w_{M1} & w_{M2} & \cdots & w_{ML-1} \end{array}]$ represents the weight matrix between the input layer and the variable hidden layer. *B* represents the threshold matrix of the hidden layer neuron node. $B={\left[b_{1},b_{2},\ldots ,b_{L-1}\right]}^{T}$, where *b*_*j*_ represents the threshold of the *j* hidden layer neuron. *g*(•) is the activation function of the hidden layer neuron node, the *sigmoid* function is the hidden layer neuron activation function of the SCNs, and $g_{j}=[g_{j1},g_{j2},\ldots ,g_{jN}]$ is the output of the *j* hidden layer neuron.

$$g_{j}=g(w_{j}^{T}X+b_{j})=\frac{1}{1+\exp(-w_{j}^{T}X+b_{j})},$$ (1)

where *w*_*j*_ is the connection weight between the input node and the *j* hidden node. The output matrix of the hidden layer node of the network are $H={\left[g_{1},g_{2},\ldots ,g_{L-1}\right]}^{T}=[\begin{array}{cccc} g_{1,1} & g_{1,2} & \cdots & g_{1,N}\\ g_{2,1} & g_{2,2} & \cdots & g_{2,N}\\ \vdots & \vdots & \ddots & \vdots \\ g_{L-1,1} & g_{L-1,2} & \cdots & g_{L-1,N} \end{array}]$. *β* are the connection weight matrix between the hidden layer and the output layer. $\beta =[\begin{array}{cccc} \beta_{1,1} & \beta_{1,2} & \cdots & \beta_{1,K}\\ \beta_{2,1} & \beta_{2,2} & \cdots & \beta_{2,K}\\ \vdots & \vdots & \ddots & \vdots \\ \beta_{L-1,1} & \beta_{L-1,2} & \cdots & \beta_{L-1,K} \end{array}]$, where *β*_*jk*_ is the connection weights between the *j* hidden node and the *k* output node. $O={\left[O_{1},O_{2},\ldots ,O_{K}\right]}^{T}$ is the actual output of the network. The output of the stochastic configuration networks with the *M*−(*L*−1)−*K* structure is expressed as

$$O=\beta^{T}H.$$ (2)

Given an objective function $f:{ℜ}^{M}\to {ℜ}^{K}$, the output of the current network is.


$$f_{L-1}(X)=\beta^{T}H=\overset{L-1}{\underset{j=1}{\sum }}\beta_{j}g_{j}(w_{j}^{T}X+b_{j})(L=1,2,\ldots ;f_{0}=0).$$ (3)

The residual of the current network is

$$e_{L-1}=f-f_{L-1}=[e_{L-1,1},e_{L-1,2},\ldots ,e_{L-1,K}].$$ (4)

If the output residual *e*_*L*−1_ of the current network does not reach the ideal error set of this network in advance, then the network will gradually increase the number of neurons in the hidden layer. Subsequently, the appropriate range of random parameters is selected automatically according to the inequality constraints to obtain the *L* hidden layer node parameters, i.e., *g*_*L*_ (*w*_*L*_ and *b*_*L*_).

The inequality constraint for obtaining the hidden layer node parameters in the SCNs is

$$\xi_{L,K}=(\frac{{\left(e_{L-1,k}{\left(X\right)}^{T}•g_{L}(X)\right)}^{2}}{g_{L}{\left(X\right)}^{T}•g_{L}(X)}-(1-r-\mu_{L})e_{L-1,k}{\left(X\right)}^{T}•e_{L-1,k}(X))\geq 0,$$ (5)

where $g_{L}=g_{L}(w_{L}^{T}X+b_{L})$ represents the output of the *L* hidden layer neuron, and *e*_*L*−1_ is the output residual of the stochastic configuration networks when constructing the *L*−1 hidden layer neuron. In 0<*r*<1, the value can be determined according to the process of hidden layer node parameter selection. When appropriately adjusted, $\mu_{L}=\frac{1-r}{L+1}$ is a sequence of nonnegative real num *e*_*L*_ bers. On the basis of the inequality constraints of Eq (5), we can therefore conclude that the distribution of the input training samples has a direct impact on the threshold selection and parameter weights of the stochastic configuration networks.

The output weight of the *L* hidden layer neuron is obtained via Eq (7).

$$\beta_{L,K}=\frac{\langle e_{L-1,K},g_{L}\rangle }{{\left\|g_{L}\right\|}^{2}},k=1,2,\ldots ,K.$$ (6)

When the *L* hidden layer neuron is constructed, the output of the stochastic configuration networks is

$$f_{L}(X)=f_{L-1}(X)+\beta_{L}g_{L}(w_{L}^{T}X+b_{L}).$$ (7)

**Fig 5:**
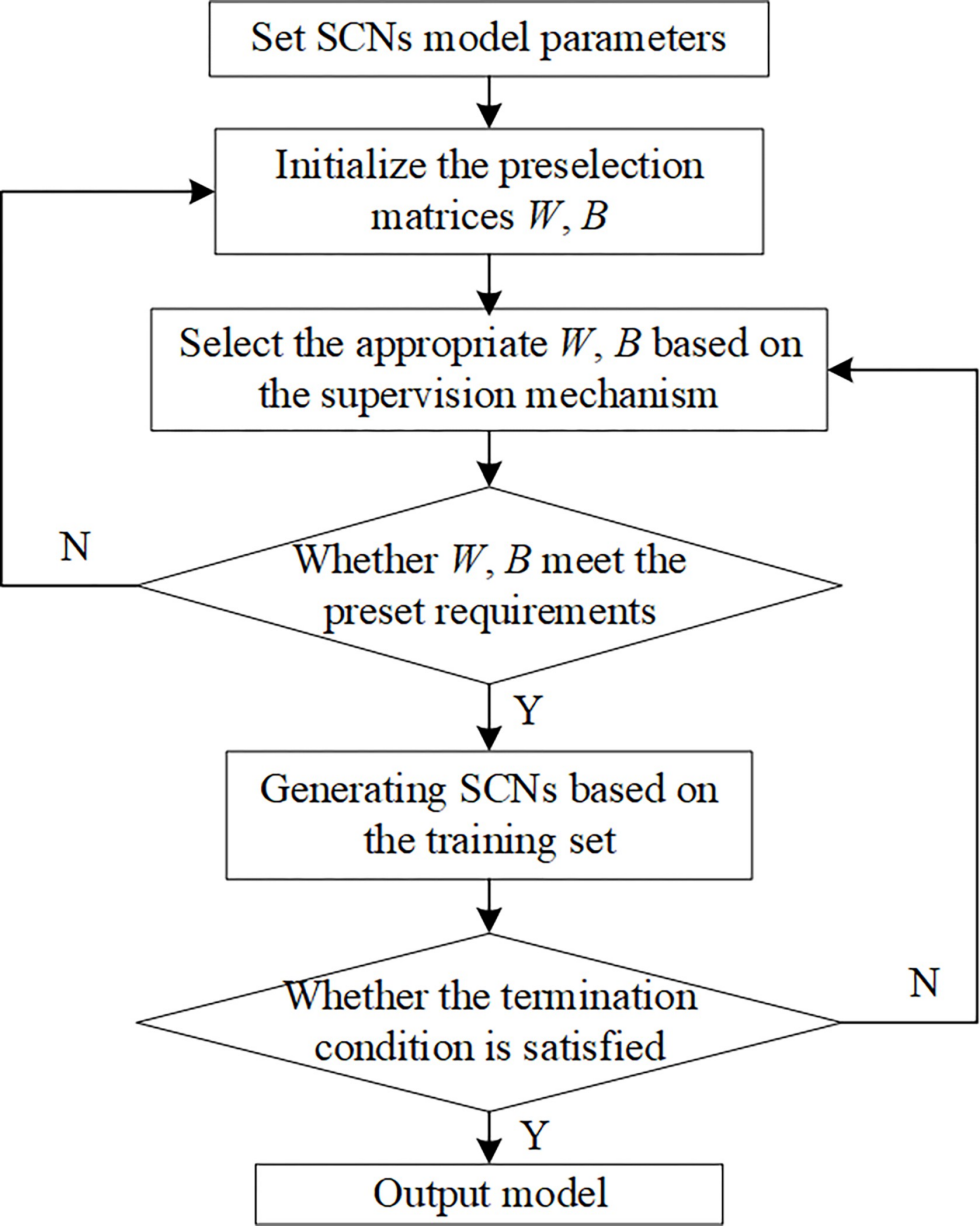
Basic flowchart of SCNs.

We obtain the output of the stochastic configured networks and then judge whether the residual *e*_*L*_ of the network output would meet the preset requirements for the error. If the output residual meets the preset requirements, then the SCNs model is constructed. If the output residual does not meet the preset requirements, then we continue to follow the inequality constraints shown in Eq (6) and add hidden nodes to reduce the residual error of the network output until the residual error of the output meets the preset. Then, we stop the training and output the SCNs model. The basic flowchart of the SCNs is shown in Fig 5.

#### 3.2.3 Establishment of the Bagging-SCNs short-term load forecasting model

**Fig 6:**
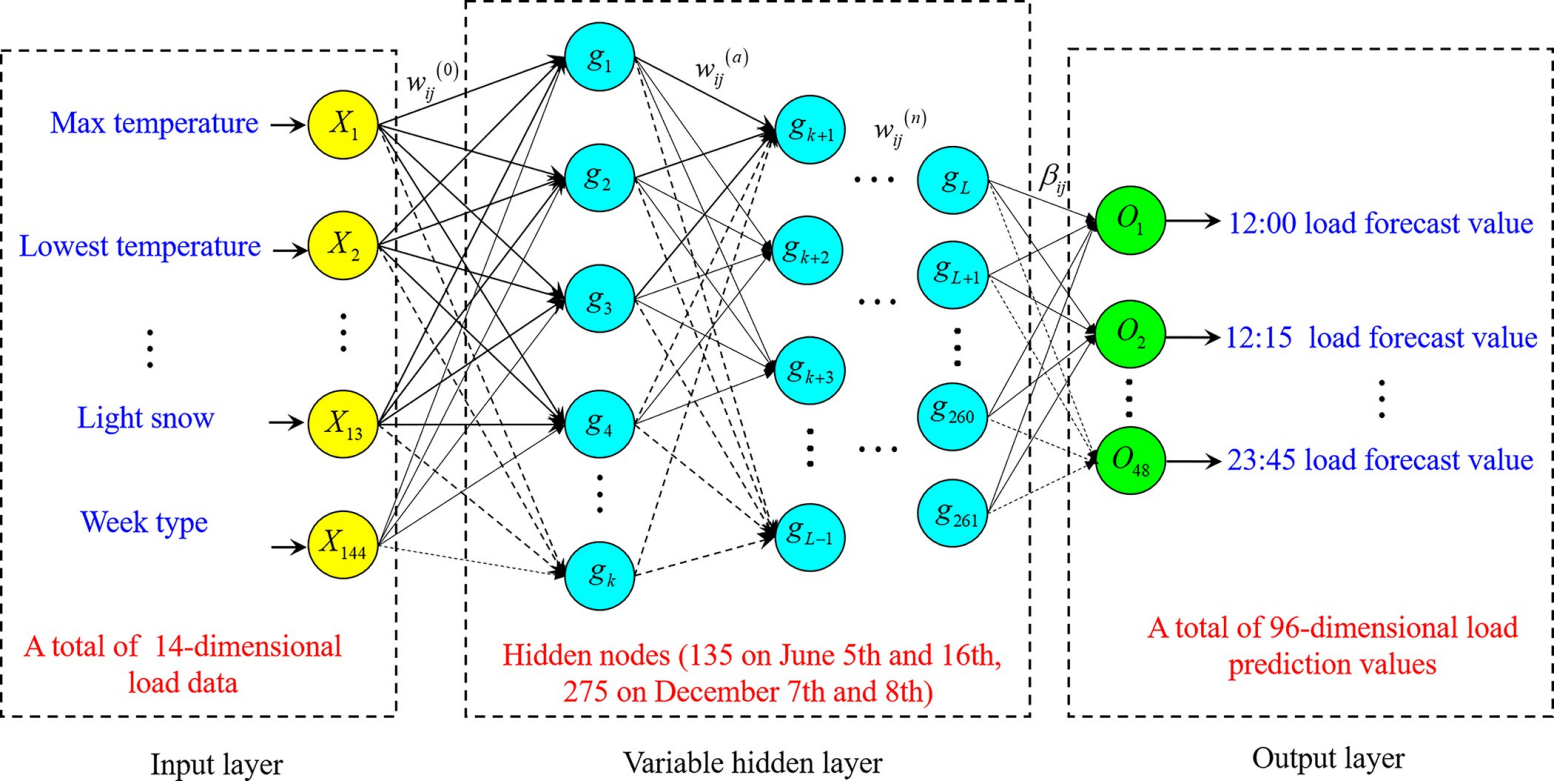
Short-term load forecasting model of SCNs.

The construction of the SCNs model is shown in Fig 6. By using encoded weather, weekly type, maximum temperature, and minimum temperature data from January 1, 2018 to the day before the forecast date, four validation groups (June 15, June 16, December 7, and December 8) can input 166, 167, 348 and 349 influencing factors, respectively, with 14 dimensions of influencing factors. The input layer has 14 neurons, the hidden layer has 135, 135, 275 and 275 neurons respectively, and the output layer has 96 neurons.

The idea of ensemble learning is to combine multiple basic learners to solve the same problem for improving the performance of the integrated model. According to the basic principles of the Bagging algorithm introduced above, the Bagging ensemble learning initially trains each base learner through different subsets of the training samples and then combines the trained base learners through a certain combination strategy as a means of combining individually weak learners. In this manner, an ensemble of strong learners can be achieved.

The performance of multiple unstable base learners integrated by Bagging is much higher than that of a single unstable base learner; on the basis of this idea, the generalization ability of the model can be greatly improved. SCNs have the advantage of fast learning speed, can automatically generate a corresponding network structure according to the characteristics of input data, have strong approximation ability to input data, and can effectively reduce errors caused by human intervention. The subset of training samples generated by Bootstrap resampling is used to train each base learner. Hence, the difference between each base learner can be increased, and the generalization performance of the integrated model can be further improved. From these combined advantages and disadvantages of the Bagging and SCNs algorithms, a short-term load forecasting method of Bagging-SCNs is proposed.

**Fig 7:**
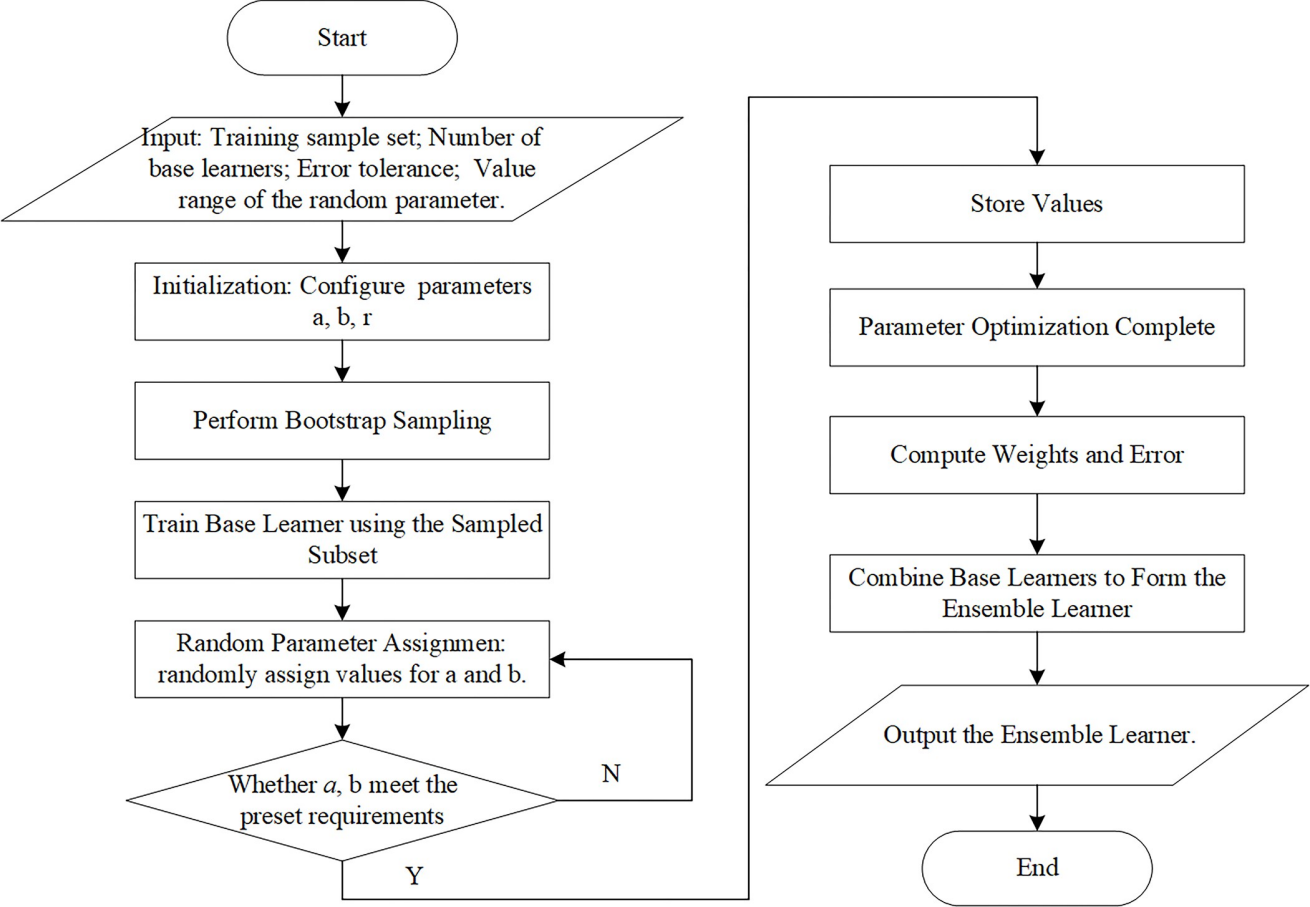
Forecasting process of Bagging-SCNs.

The basic flowchart of the Bagging-SCNs is shown in Fig 7. First, the original training data are divided to obtain a training set and a test set, and then the training set is resampled by the Bootstrap method to obtain mutually different training subsets. Then, SCNs are selected as the base learner for each training subset for training. Finally, the output of all single SCNs base learners is averaged as the final output of the model, and the prediction effect of the Bagging-SCNs algorithm is evaluated through the test set.

The steps of the algorithm are as follows:

**Algorithm:** Bagging-SCNs

**Input:** Given training sample set $X=\{X_{1},X_{2},\ldots ,X_{N}\}$; The expected output for the corresponding sample $T=\{t_{1},t_{2},\ldots ,t_{N}\}$; Number of base learners *P*;The maximum number of neurons in the hidden layer of SCNs *L*_max_;Error tolerance *ε*;The size of the preselection matrix *T*_max_;The value range of the random parameter ϒ = {*λ*_min_,…,*λ*_max_};

**Output:** An ensemble learner *R*(*X*)

1: **Initialization:** $e_{0}={\left[t_{1},t_{2},\ldots ,t_{N}\right]}^{T}$, **0<*r*<1**, Ω, *W* = ∅;

2: **for *I* = 1** to *P* **do**

3: Use the Bootstrap method to extract a training subset *X*_*I*_ of the same size as *X* from the training sample set *X* with replacement;

4: Train the corresponding base learner *θ*_*I*_(*x*) based on different training subsets *X*_*I*_;

5: **while** *L*≤*L*_max_ and ‖*e*_0_‖_*F*_>*ε*, **do**

6: **for** *λ*∈ϒ, **do**

7: **for** *k* = 1,2,…,*T*_max_, **do**

8: randomly assign *w*_*L*_and *b*_*L*_ from intervals [−*λ*,*λ*]^*M*^ and [−*λ*,*λ*], respectively;

9: calculate $g_{L}(X)=[g_{L}(w_{L}^{T}x_{1}+b_{L}),g_{L}(w_{L}^{T}x_{2}+b_{L}),\ldots ,g_{L}(w_{L}^{T}x_{N}+b_{L})]$;

10: calculate $\xi_{L,K}=(\frac{{\left(e_{L-1,k}{\left(X\right)}^{T}•g_{L}(X)\right)}^{2}}{g_{L}{\left(X\right)}^{T}•g_{L}(X)}-(1-r-\mu_{L})e_{L-1,k}{\left(X\right)}^{T}•e_{L-1,k}(X))$;

11: calculate $\mu_{L}=(1-r)/(L+1)$;

12: **if** $\min\{\xi_{L,1},\xi_{L,2},\ldots ,\xi_{L,K}\}\geq 0$

13: store *w*_*L*_ and *b*_*L*_ in *W* and $\xi_{L}=\sum_{k=1}^{K}\xi_{L,k}$ in Ω respectively;

14: **else**

15: go to **step 7**

16: **end if**

17: **end for**

18: **if** *W* is not an empty set

19: find $w_{L}^{*}$ and $b_{L}^{*}$ in Ω that maximize *ξ*_*L*_, and then get matrix $\left[g_{1}^{*},g_{2}^{*},\ldots ,g_{L}^{*}\right]$;

20: **Break** (go to **step 24**)

21: **else**

22: randomly take *τ*∈(0,1−*r*), update *r*: *r* = *r*+*τ*, go back to **step 7**

23: **end if**

24: **end for**

25: calculate the optimal output weights

$\left[\beta_{1}^{*},\beta_{2}^{*},\ldots ,\beta_{L}^{*}]=\underset{\beta }{\arg\;\min}\|f-\sum_{j=1}^{L}\beta_{j}g_{j}(w_{j}^{T}X+b_{j})\right\|$

26: calculate output error $e_{L}=e_{L-1}-\beta_{L}^{*T}g_{L}^{*}$;

27: update *e*_0_:*e*_0_ = *e*_*L*−1_; *L*: *L* = *L*+1;

28: **end while**

29: **end for**

30:**Return** $\beta_{1}^{*},\beta_{2}^{*},\ldots ,\beta_{L}^{*}$; $w^{*}=[w_{1}^{*},w_{2}^{*},\ldots ,w_{L}^{*}]$; $b^{*}=[b_{1}^{*},b_{2}^{*},\ldots ,b_{L}^{*}]$

31: Combine *P* basic learner model *θ*_*I*_(*x*) to get an integrated learner $R(X)=\frac{1}{P}\sum_{I=1}^{P}\theta_{1}(x)$

## 4 Example analysis

### 4.1 Data source

**Fig 8:**
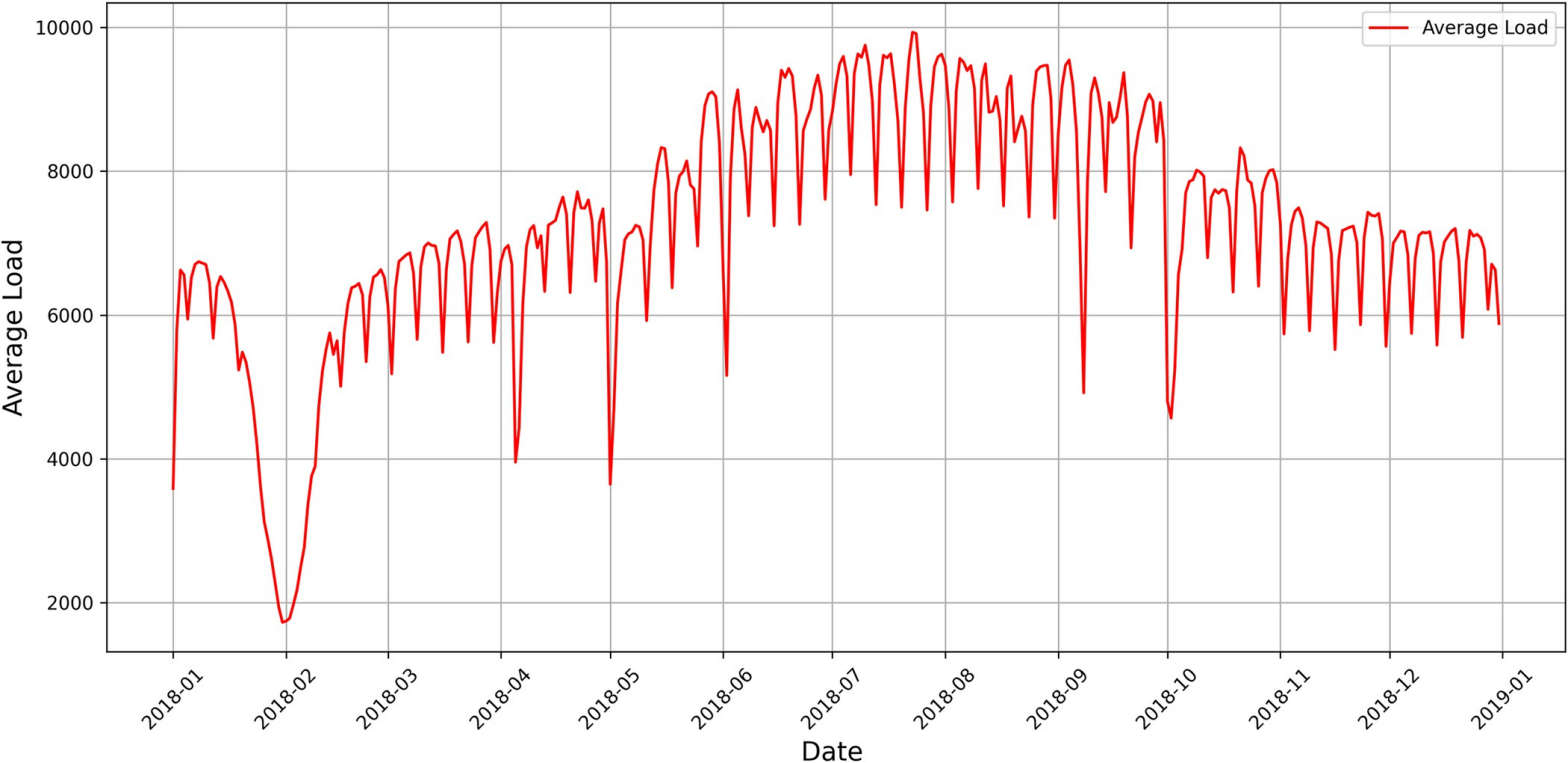
Time-dependent change of average load.

**Fig 9:**
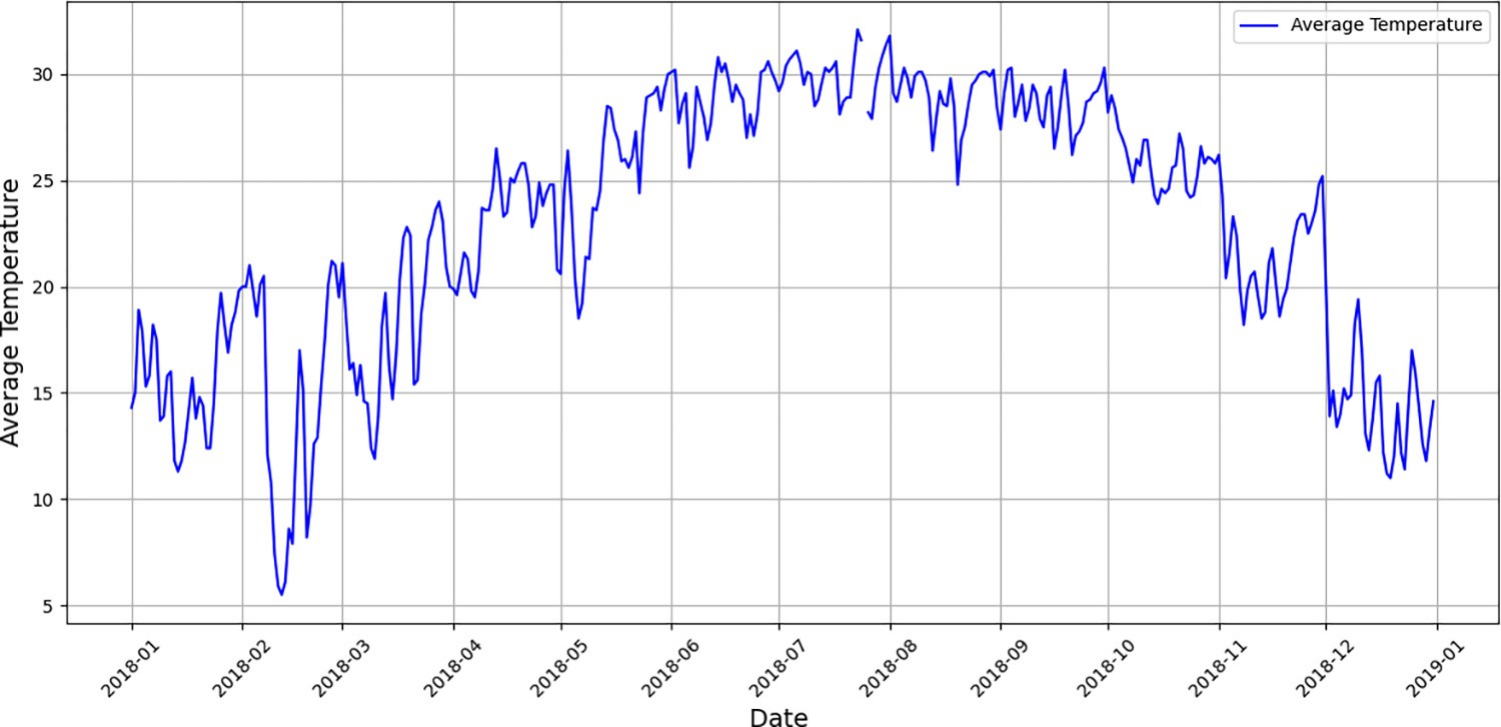
Time-dependent change of average temperature.

The data used in this study are the daily load data of Quanzhou City, Zhejiang Province from January 1, 2018 to December 31, 2018, which covers 365 days. From 0:00 to 23:45 every day, the data are collected every 15 minutes, with a total of 96 sampling points per day. For the influencing factors of the short-term load, this research mainly considers weather, maximum temperature, minimum temperature, week type, and time span from January 1, 2018 to December 31, 2018. After coding the weather, this parameter is decomposed into sunny, cloudy, overcast, fog, thunderstorm, light rain, moderate rain, heavy rain, heavy rain, sleet, and light snow. The specific coding method has been introduced in detail in Section 3.1. Through this decomposition, the original one-dimensional weather data can be expanded into 11-dimensional weather data, and then the influencing factors are expanded from 4-dimensional to 14-dimensional data. The complete data for average load and average temperature is shown in Figs 8 and 9.

### 4.2 Short-term load forecasting

**Fig 10:**
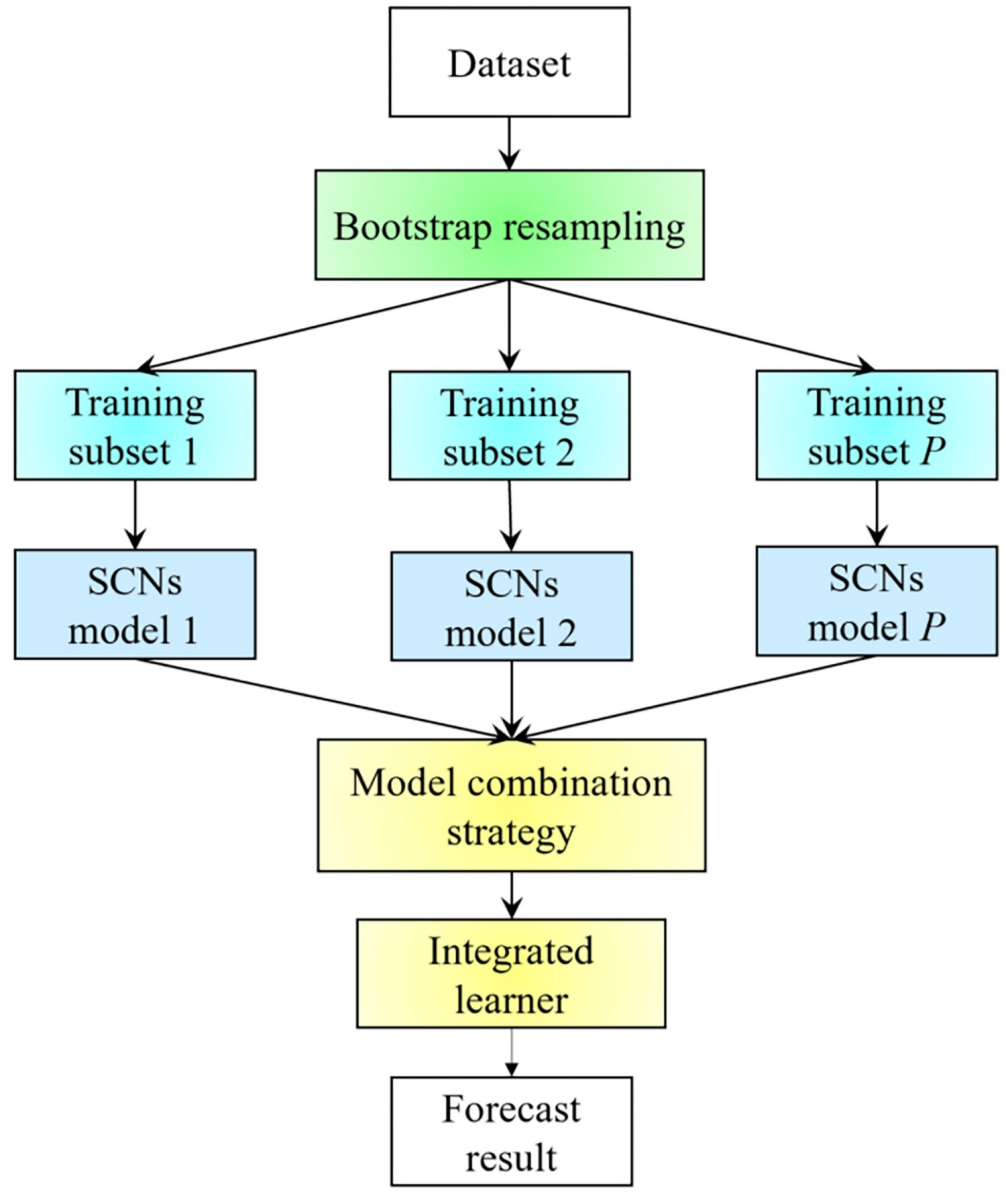
Forecasting process of Bagging-SCNs.

Aiming to verify the effectiveness of the proposed method based on Bagging-SCNs for daily load forecasting, daily load data collected every 15 minutes from January 1, 2018 to the day before the forecast date were used as the training set. In particular, the Bagging-SCNs algorithm is used to establish a short-term load forecasting model for forecasting the daily load data on June 15th, June 16th, December 7th, and December 8th. Through data preprocessing, the missing data in the training set are filled with the average value, and the weather and week-type data are encoded. The weather variable is converted into an 11-dimensional matrix. For the training set whose data have been preprocessed, it is resampled by the Bootstrap method to obtain multiple training subsets that differ from one another, and then SCNs are selected as the base learner for each training subset for training. The output of the learner is averaged as the final output of the model, and the prediction effect of the Bagging-SCNs algorithm is evaluated via the test set. The proposed algorithm is programmed in Python language. The short-term load forecasting process is shown in Fig 10.

The error evaluation indicators include the mean absolute percentage error (MAPE) and root mean-square error (RMSE). The formula for calculating MAPE is

$$\mathrm{MAPE}=\frac{1}{n}\overset{n}{\underset{i=1}{\sum }}|\frac{{\hat{y}}_{i}-y_{i}}{y_{i}}|\times 100\%,$$ (8)

and the formula for calculating RMSE is

$$\mathrm{RMSE}=\sqrt{\frac{1}{n}\overset{n}{\underset{i=1}{\sum }}{\left({\hat{y}}_{i}-y_{i}\right)}^{2}},$$ (9)

where *y*_*i*_ is the actual value of the *i* sample, ${\hat{y}}_{i}$ is the predicted value of the *i* sample, and *n* is the number of sample points.

Aiming to verify the effectiveness of the proposed method, we use 135, 135, 275, 275 hidden layer neurons for Bagging-SCNs, SCNs, and LSTM for four forecast days separately. The specific parameters of SCNs are as follows: error tolerance *tol* of 0.001; maximum number of random

Configurations *T*_max_ of 100; maximum number of hidden nodes *L*_max_ of 300; and random weight range *Lambdas* of [0.1,0.15,0.2,…,10]. All three models are evaluated based on the mean absolute percent error of prediction.

**Fig 11:**
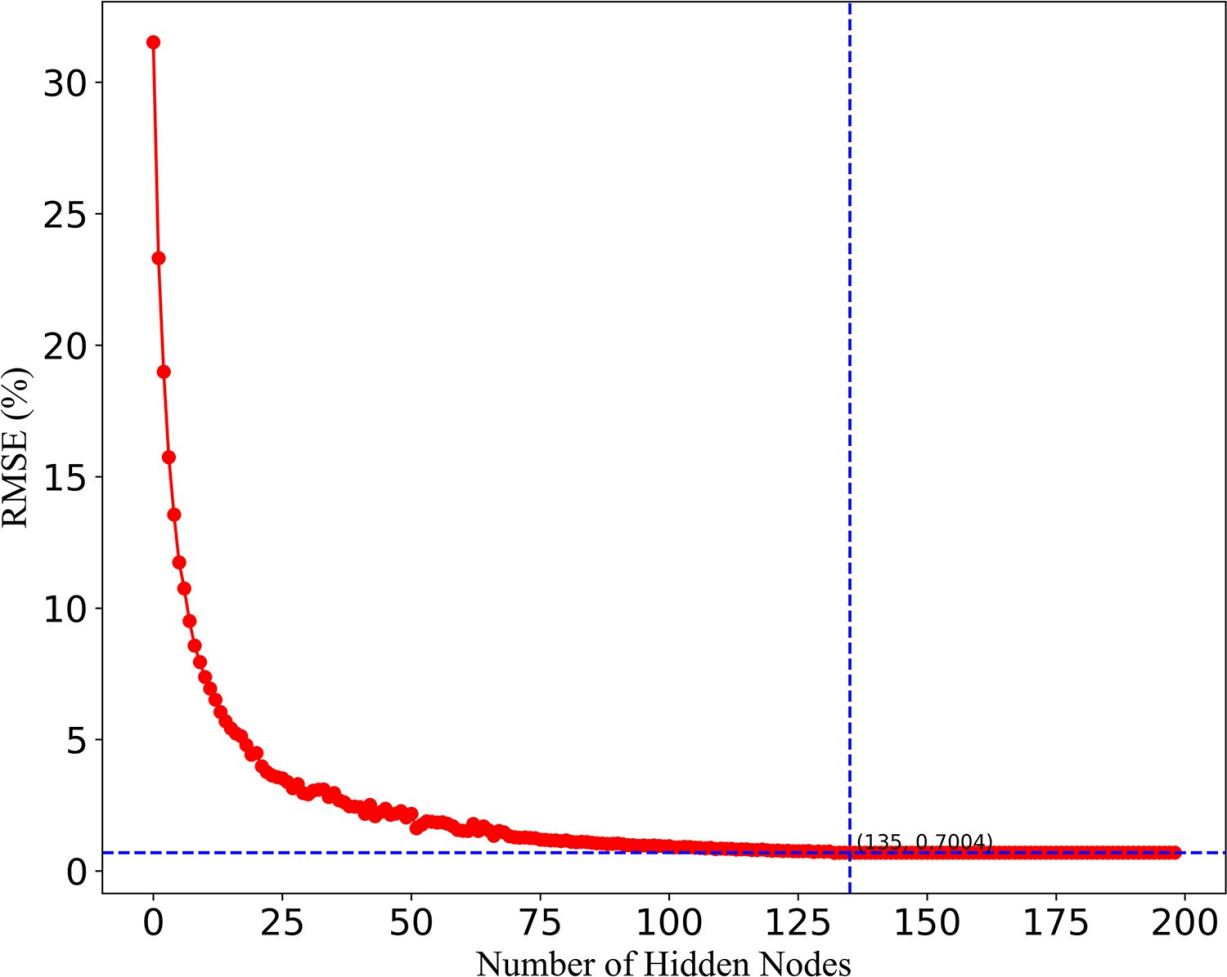
Relationship between number of hidden nodes of SCNs and training error.

When performing daily load forecasting, each basic learner in the SCNs should be trained initially, and RMSE is adopted as the training standard. To present the relationship between the number of hidden layer neurons in the basic learner and the model training error, we take the prediction results on June 15th as an example, as shown in Fig 11 and Table 1. As the number of neurons in the hidden layer increases, the training error (RMSE) of SCNs first decreases sharply, then decreases slowly, and finally stabilizes. The reason for the stabilization is that the preset accuracy requirement is reached at this time. The SCNs stop the training When the number of hidden nodes reaches 135, 135, 275, 275 on the four predicted days mentioned above, and the preset model (i.e., 300 hidden nodes) is not reached. At this time, the training errors (RMSE) are 0.0324, 0.0443, 0.0440, and 0.0406, respectively.

**Table 1:** Number of hidden nodes of SCNs and the corresponding training error.

Number of hidden nodes	RMSE%	Number of hidden nodes	RMSE%	Number of hidden nodes	RMSE%	Number of hidden nodes	RMSE%	Number of hidden nodes	RMSE%
1	47.290	11	7.952	140	0.700	160	0.700	193	0.700
2	31.527	12	7.377	141	0.700	161	0.700	194	0.700
3	23.316	13	6.939	142	0.700	162	0.700	195	0.700
4	18.992	14	6.521	143	0.700	163	0.700	196	0.700
5	15.738	15	6.051	144	0.700	164	0.700	197	0.700
6	13.552	⋯	⋯	⋯	⋯	⋯	⋯	198	0.700
7	11.736	134	0.689	156	0.700	179	0.700	199	0.700
8	10.744	135	0.700	157	0.700	180	0.700	200	0.700

**Fig 12:**
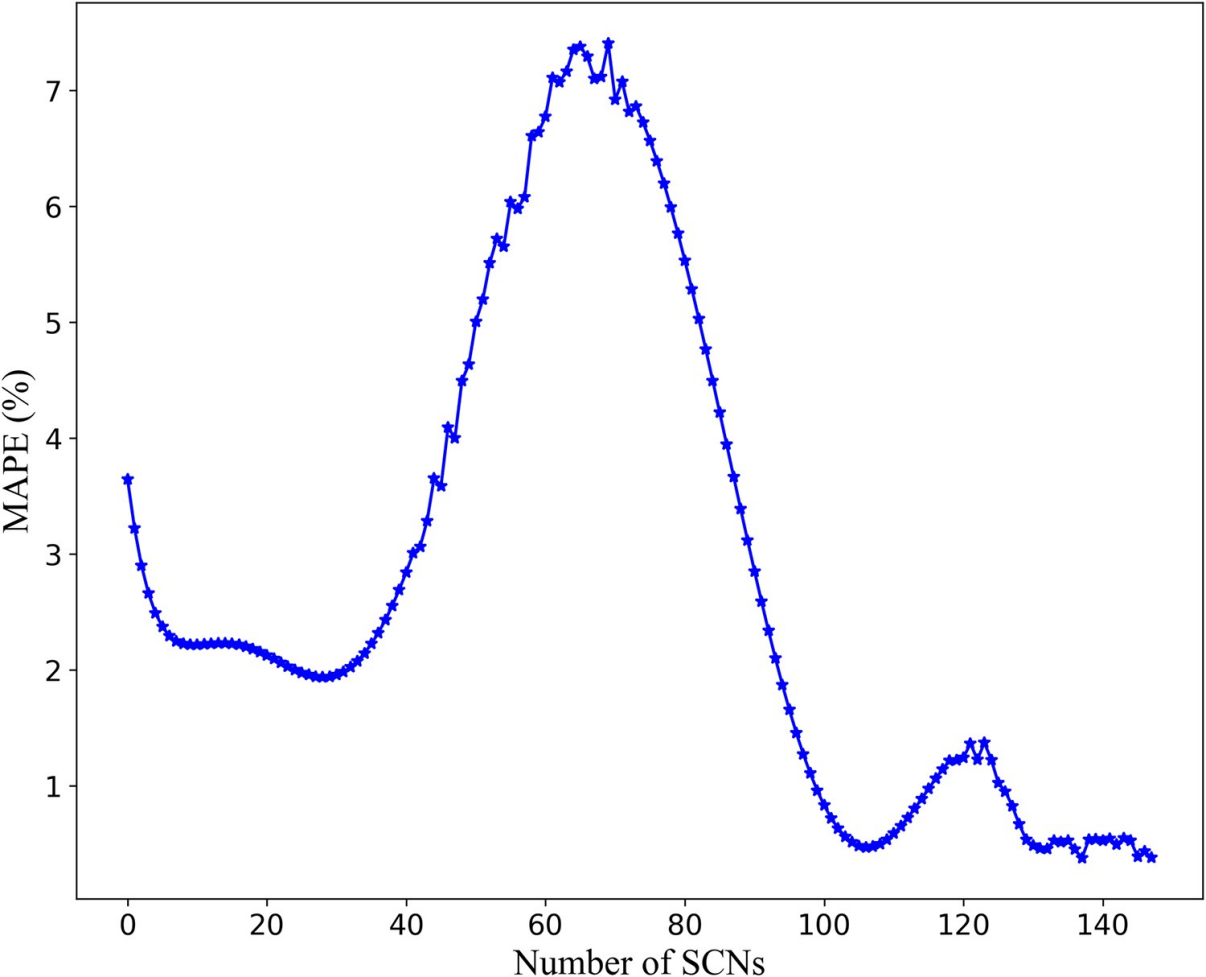
Relationship between number of SCNs-based learners and error.

The relationship between the number of basic learners in SCNs and the model output error is shown in Fig 12 and Table 2. As shown in Fig 12, when the number of SCNs is around 8, the average absolute percentage error of the model is around 2.37% and tends to be stable. However, when the number of SCNs base learners is large, the calculation amount will increase rapidly, and the prediction time will be longer. In view of comprehensively considering modeling speed and prediction error, this study selects 60 SCNs base learners to build the Bagging-SCNs prediction model. Fig 12 also shows that SCNs can meet the preset accuracy requirements when the number of hidden nodes is 135. Thus, the number of hidden layer neurons of each base learner is set to 135, and the maximum number of preselected neurons is set to 100. In addition, the hidden nodes of the base learner in SCNs are added only one at a time.

**Table 2:** Number of SCNs-based learners and corresponding error.

Number of SCNs	MAPE%	Number of SCNs	MAPE%	Number of SCNs	MAPE%	Number of SCNs	MAPE%	Number of SCNs	MAPE%
1	4.886	11	2.225	51	4.495	91	3.392	141	0.559
2	4.192	12	2.217	52	4.639	92	3.119	142	0.540
3	3.645	13	2.217	53	5.005	93	2.852	143	0.486
4	3.221	14	2.222	54	5.197	94	2.592	144	0.540
5	2.900	15	2.228	55	5.511	95	2.341	145	0.433
6	2.661	⋯	⋯	⋯	⋯	⋯	⋯	146	0.509
7	2.490	47	3.655	87	4.496	137	0.453	147	0.444
8	2.372	48	3.588	88	4.222	138	0.476	148	0.413

### 4.3 Analysis of prediction results

**Fig 13:**
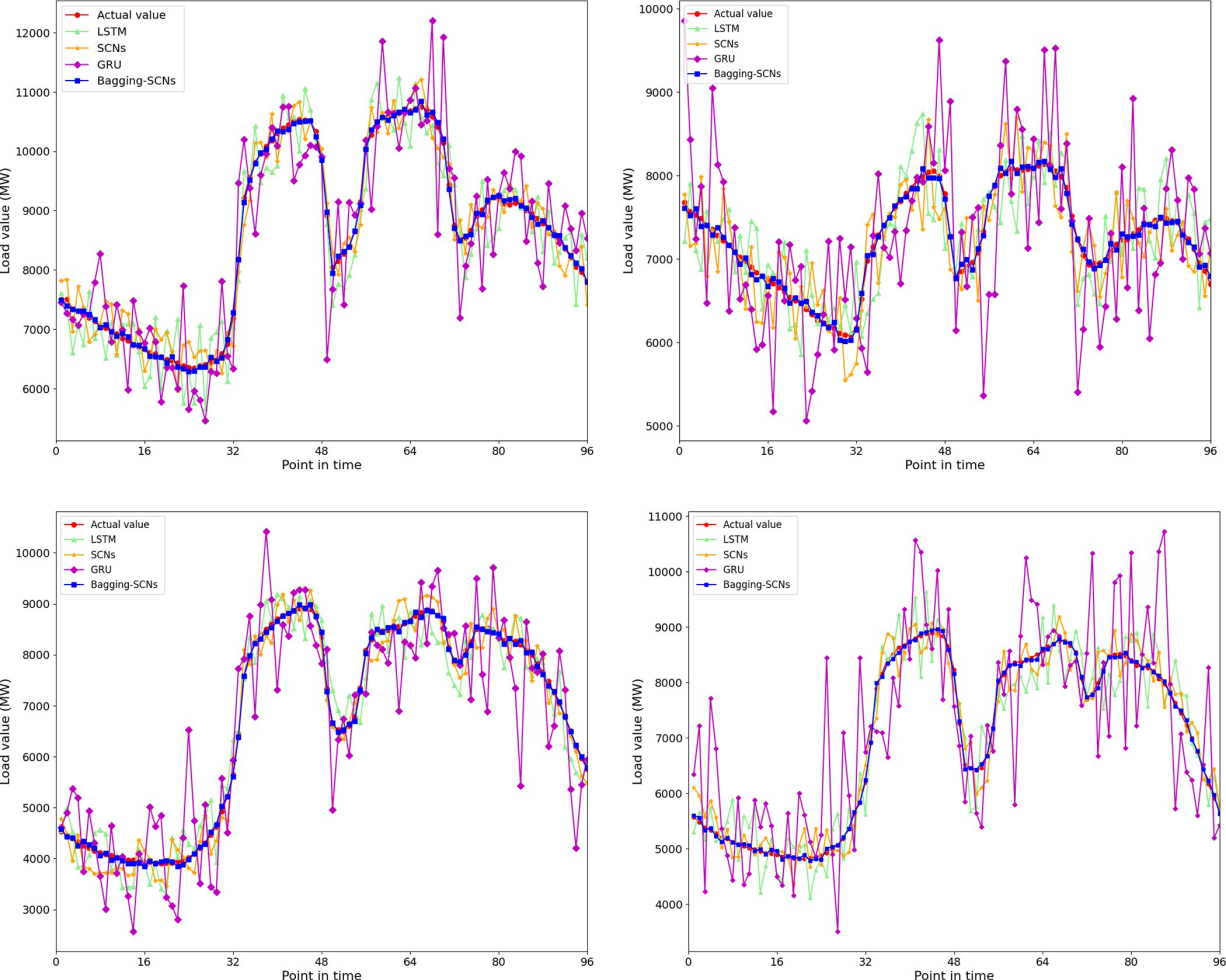
True value and predicted value of each method. (a) The predicted results for June 15, 2018. (b) The predicted results for June 16, 2018. (c) The predicted results for December 7, 2018. (d) The predicted results for December 8, 2018.

After the model is trained, the model is evaluated on the test set. The week-type data on forecast day (June 15, June 16, December 7, December 8, 2018), the encoded weather data, the highest temperature, the lowest temperature, and the 165, 166, 348, 349 14-dimensional data are taken as the input of the prediction. The output is forecast day 0:00 to 23:45 96 daily load forecast data at 15-minute intervals. Then, this study uses the LSTM neural network, SCNs, Bagging-SCNs for the algorithm programming in Python language and for daily load forecasting. The predicted results corresponding to the forecast days (June 15, June 16, December 7, December 8, 2018) are shown in Fig 13(A)-13(D).

As shown in Fig 13, the actual daily average load on June 15, June 16, December 7, and December 8, 2018 are 8570.87MW, 7240.27MW, 6737.63MW, and 7014.67MW, respectively. The daily average load predicted by the LSTM neural network are 8505.64 MW, 7272.51MW, 6731.43MW and 7026.81MW, respectively. And the MAPE are 4.76%, 4.89%, 5.77%, 5.81%, respectively. Furthermore, the daily average load predicted by the GRU neural network are 8567.09MW, 7256.71MW, 6639.54MW and 7201.93MW, respectively, with a Mean Absolute Percentage Error (MAPE) of 6.59%, 9.39%, 11.61% and 13.57%. Although the LSTM neural network and the GRU neural network can perform well in terms of daily average load forecasting, the forecasting effect at individual time points is not ideal, and the predicted value has a low degree of agreement with the actual value. Nonetheless, the overall trend of the predicted value is close to that of the actual value. The individual prediction errors are mainly concentrated in the middle part. The large error of the prediction value corresponding to the time point in the middle part can be attributed to the gradient explosion during the prediction, which reduces the prediction accuracy.

The daily average load predicted by SCNs are 8606.41MW, 7209.91MW, 6688.27MW and 7057.04MW, respectively, and the MAPE are 3.24%, 4.43%, 4.40% and 4.06%, respectively. Although SCNs are more accurate in forecasting the average daily load (the error is less than 4.5%), the predicted value at each time point has a low degree of agreement with the actual value, and the fluctuation range is large. However, the overall trend of the predicted value is close to the actual value. This phenomenon can be explained by the large number of neurons in the hidden layer, which complicates the SCNs structure and causes an overfitting. Therefore, in view of the unsatisfactory prediction results of the single-SCNs algorithm, we further use Bagging-SCNs to improve the model’s generalization performance and the accuracy of the short-term load forecasting.

**Fig 14:**
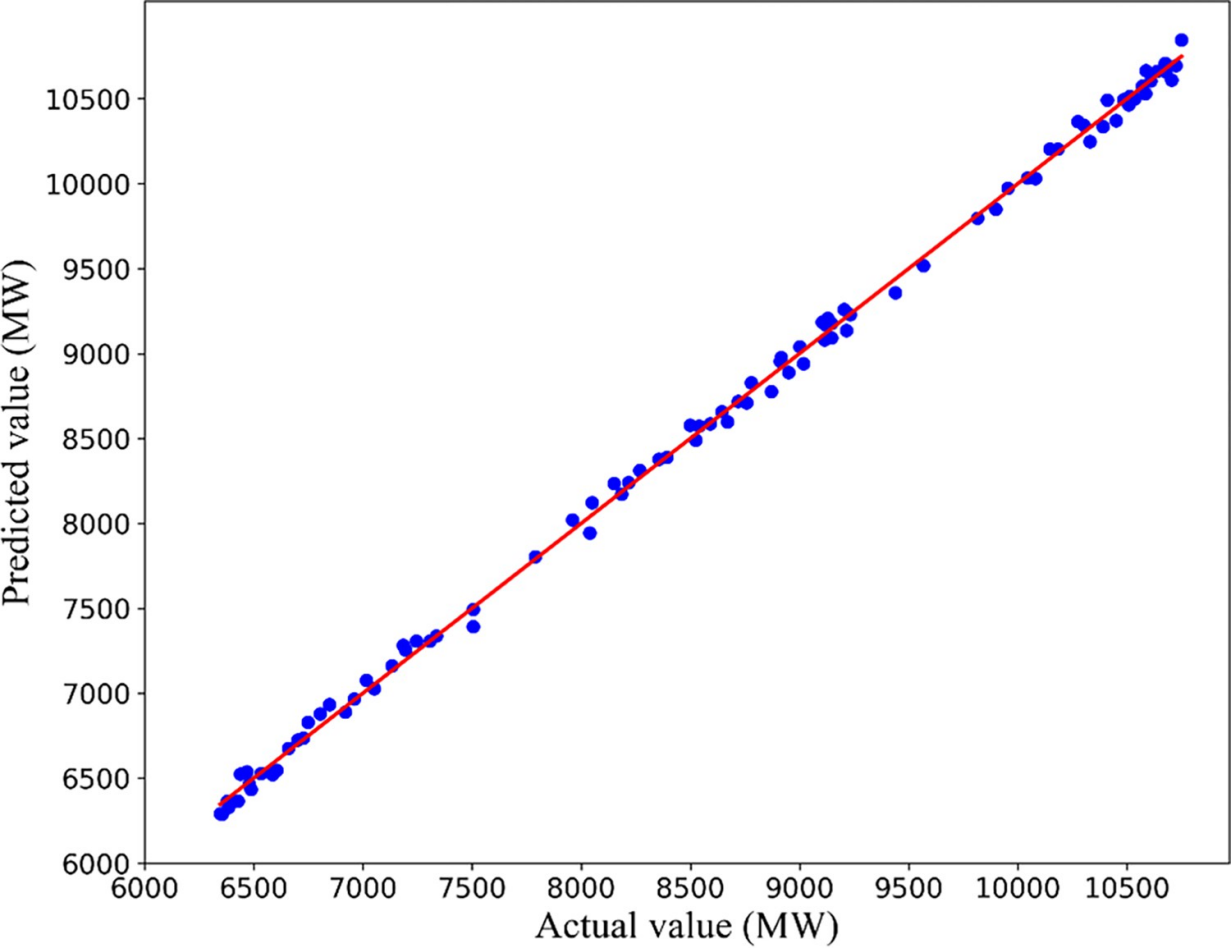
Actual value and predicted value of Bagging-SCNs.

The daily average load predicted by Bagging-SCNs are 8573.31MW, 7236.44MW, 6733.13MW and 7018.13MW, and the MAPE are 0.54%, 0.69%, 0.75% and 0.64%. The Bagging-SCNs algorithm is highly accurate in forecasting the daily average load (the error is less than 1%). Furthermore, the predicted value at each time point is highly consistent with the actual value, and the overall trend of the predicted value is much closer to the actual value. The scatter plot for the predicted and actual values obtained by Bagging-SCNs is shown in Fig 14.

The diagonal line on the scatterplot indicates that the predicted value is equal to the actual value. The higher the prediction accuracy, the closer the point to the diagonal line. The predicted value is close to the actual value, and the predicted value is mostly concentrated near the diagonal line. However, the predicted and actual values have a large deviation at individual time points. In contrast to the LSTM neural network and the GRU neural network, Bagging-SCNs can improve MAPE by at least 4.20% and 6.05%, respectively. Compared to SCNs, it shows an improvement of at least 2.70%. The experimental results indicate that Bagging-SCNs can obtain better prediction results.

## 5 Conclusion

In terms of short-term load forecasting, the traditional short-term load forecasting method has limited nonlinear mapping ability and weak generalization ability to unknown data. Thus, this study proposes a short-term load forecasting method based on Bagging-SCNs. First, the training set is resampled via the Bootstrap method to obtain multiple mutually different training subsets, and then SCNs are selected as the base learner for each training subset. After training, the output of all single-SCNs base learners is averaged as the final output of the model. The accuracy and effectiveness of the proposed method have been proven by practical examples. The main conclusions of this study can be summarized as follows.

1. When the data sample is large because of the gradual increase in the number of neurons in the hidden layer of a single-SCNs, the network structure of the SCNs will become more complex, and the model is prone to overfitting. The Bagging algorithm can integrate multiple SCNs to reduce the variance of the model and improve its generalization performance while effectively improving the prediction accuracy.
2. The prediction content can be completed at a lower cost in terms of training time. This method requires less parameters to be manually determined, and the model has a higher degree of intelligence, suggesting its feasibility as a method. Thus, more efficient prediction methods can be better applied to the operational decision making of smart distribution networks.

This study has only considered the four influencing factors of week type, weather, maximum temperature, and minimum temperature. Holiday factors also affect short-term load forecasting. Future researchers may increase the collection of datasets, analyze and process each of the influencing factors of holiday loads, and conduct short-term load forecasting based on Bagging-SCNs while considering the holiday loads.

## Supporting information

S1 Data (ZIP)
