# Supplementary material for: Short-term power load forecasting method based on Bagging-stochastic configuration networks
Source: PLoS One. 2024 Mar 19;19(3):e0300229. doi: 10.1371/journal.pone.0300229 (PMC10950235; doi:10.1371/journal.pone.0300229)
Supplement: S1 Data — (ZIP) [file pone.0300229.s001.zip › Data Availability Statement/Data Availability Statement.docx]

**Data Availability Statement**

In this article, I have referenced three Excel tables, and the following are data descriptions of their contents.

Input Table (Figures 8 and 9): Data source for short-term load forecasting. excel. The table size is 366 * 101. The first column is the date, the second to ninety-seven columns are the power load values at 96 times of the day, the ninety-eight columns are the week, the ninety-nine columns are the weather, and the 100th column is the maximum temperature. Column 101 is the minimum temperature.

Table 1: Number of hidden nodes of SCNs and the corresponding training error. excel. The table size is 200 * 2. The first column data is the training error (RMSE) of SCNs, and the second column data is the number of hidden layer neurons.

Table 2: Number of SCNs-based learners and corresponding error. excel. The table size is 150 * 2, the first column of data is the number of SCNs-based learners, and the second column is the training error (RMSE) of SCNs.
